# Absence of *FGF4* Retrogene Insertion on Chromosome 18 Results in a Tall Phenotype in Grand Basset Griffon Vendéen Dogs

**DOI:** 10.3390/vetsci12090916

**Published:** 2025-09-20

**Authors:** Tendai Mhlanga-Mutangadura, Liz Hansen, Martin L. Katz

**Affiliations:** 1 Canine Genetics Laboratory, Department of Pathobiology and Integrative Biomedical Sciences, College of Veterinary Medicine, University of Missouri, Columbia, MO 65211, USA; tendai@missouri.edu (T.M.-M.); hansenl@missouri.edu (L.H.); 2 Mason Eye Institute, School of Medicine, University of Missouri, Columbia, MO 65211, USA

**Keywords:** canine, retrogene, leg length, trait selection, fibroblast growth factor

## Abstract

Every dog breed has characteristic physical features that have been fixed through numerous generations of selective breeding. For the Grand Basset Griffon Vendéen (GBGV) breed, short-leggedness has been established as the breed standard. Although breeders have been consistent in only breeding dogs that meet this standard, occasionally litters have included dogs that are significantly taller. In many dog breeds short leg length is associated with insertion of an *FGF4* retrogene (*FGF4RG*) at specific sites on either chromosome 12 (*12-FGF4RG*) and/or 18 (*18-FGF4RG*). A study was undertaken to determine whether *FGF4RG* genotype is the basis of the height difference. Of 47 GBGVs that were evaluated, 41 had leg lengths within the breed standard and 6 were significantly taller. None of the dogs had the *12-FGF4RG* insertion. The short-legged dogs were either homozygous or heterozygous for the *18-FGF4RG* insertion. All six long-legged dogs were homozygous for the non-insertion allele. This indicates that selective breeding based on phenotype alone has not been sufficient to fix short-leggedness in the breed due to retention of the non-insertion allele. The finding that one copy of the insertion is sufficient to confer short-leggedness indicates that expression of the retrogene during development is likely to inhibit growth of the limbs.

## Simple Summary

The Grand Basset Griffon Vendéen (GBGV) is one of a number of dog breeds that have been bred for a short-legged stature. Other such breeds include Dachshunds, Basset Hounds, and Corgis. Despite the fact that the GBGV breed has been propagated for many generations, short-legged parents occasionally produce litters that include both long-legged and short-legged puppies. An investigation was undertaken to determine why short-legged parents produced long-legged offspring. It was found that all of the short-legged dogs had either one or two copies of a retrogene called *18-FGF4RG*, whereas the long-legged dogs did not have any copies of this retrogene. When dogs that each had one copy of the retrogene were mated, their puppies included dogs that either had one, two, or no copies of *18-FGF4RG*. Only the puppies that had no copies had long legs. With this finding, breeders can now screen their dogs for the presence of this retrogene before mating them in order to produce puppies of the desired stature.

## 1. Introduction

Each recognized dog breed has a set of physical characteristics that have been designated as the breed standard by national and international breed organizations. Among these characteristics is height as determined primarily by leg length. A number of breeds are characterized by short stature (short-leggedness) including Dachshunds, Corgis, and Basset Hounds. Among these short-legged breeds is the Grand Basset Griffon Vendéen (GBGV). The American Kennel Club standard for this breed specifies a height of 15 to 18 inches (38 to 46 cm). The GBGV and a smaller Petit Basset Griffon Vendéen (PBGV) breed were derived from the same long-legged ancestors. Litters of the ancestral breed included smaller offspring called Petit and larger dogs called Grande [1]. Subsequent selective breeding led to the establishment of the two separate breeds, both of which have short legs relative to the related Griffon Vendéen and Grande Griffon Vendéen breeds. Canine breed-specific traits, including those for the Griffon Vendéen group of dogs, have been developed over many generations of selection for desired phenotypic features, including size [2,3,4]. Although breed standards for physical attributes continue to be based primarily on selecting dogs for breeding that exhibit the desired phenotypic features, advances in canine molecular genetics are starting to expand knowledge of the linkages between DNA sequence variants and specific phenotypic characteristics [3]. This knowledge will enable selection for specific phenotypic features based on DNA sequence analyses. Despite many generations of breeding in which the short-legged phenotype was selected for, a number of GBGV dogs that are significantly taller than the breed standard have been born recently to parents that are within the breed standard for height. Short-leggedness in a number of other breeds has been associated with *FGF4* retrogene insertions on chromosomes 12 (*12-FGF4RG*) and 18 (*18-FGF4RG*) [1,5,6,7]. In a 2009 survey of over 70 breeds, three of four GBGVs evaluated were heterozygous for the *18-FGF4RG* insertion and one was homozygous for the insertion [1]. This suggested that the presence of this insertion may be a determining factor in leg length in GBGVs. Therefore, a study was undertaken to determine whether differences in the *FGF4RG* insertion genotypes were responsible for the significant height differences among GBGVs.

## 2. Materials and Methods

### 2.1. Subject Dogs

**Figure 1:**
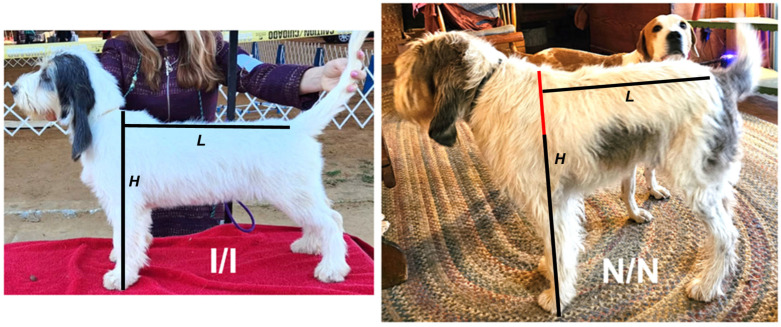
Littermate GBGVs. A dog that had the leg length typical of the breed standard (**left**) was homozygous for the *18-FGFRG* insertion (I/I). Its much taller littermate (**right**) was homozygous for the allele lacking the insertion (N/N). Height at the withers (H) was measured in each dog and body length (L) was measured as indicated. There was no difference in body length, but the N/N dog was taller by the amount indicated by the red portion of the H line.

**Figure 2:**
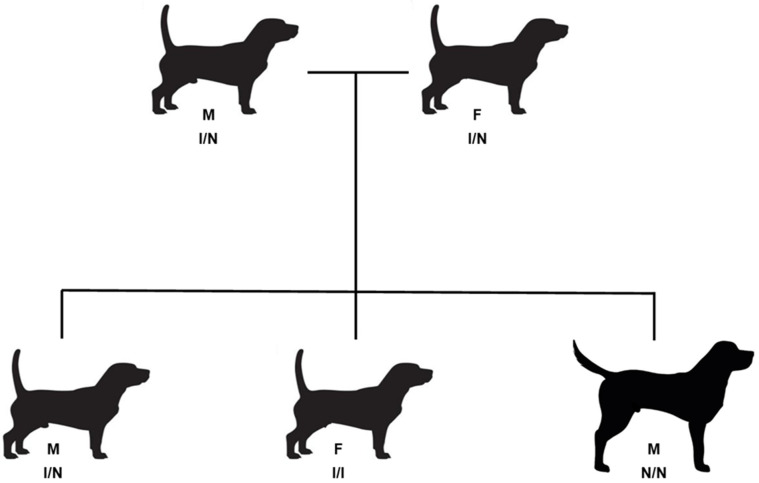
Partial GBGV pedigree that included littermates with both long and short legs. Both the female (F) and male (M) parents had the short legs characteristic of the breed and were heterozygous for the *18-FGF4RG* insertion (I/N). Offspring included puppies that were short-legged and long-legged. The short-legged dogs were either homozygous for the *18-FGF4RG* insertion (I/I) or heterozygous (I/N). The long-legged dog was homozygous for the allele lacking the insertion (N/N). All four short-legged dogs were between 39 and 45 cm tall at the withers, whereas the long-legged dog was 60 cm tall.

A total of 47 GBGVs (21 female, 26 male) born between 2010 and 2023 were evaluated for this study. Forty-one of the dogs were within the breed standard 38 to 46 cm height and six were at least 58 cm tall (Figure 1). Five of the GBGVs consisted of a mated pair of short-legged dogs and 3 of their offspring (Figure 2). The remaining tall GBGVs were offspring of 5 different additional sets of short-legged parents that were not closely related. The majority of the remaining short GBGVs were either littermates or parents of the tall GBGVs or were not closely related to the other dogs. Dog pedigree information and DNA samples from purebred GBGV dogs were provided by the dog owners and breeders. Stature of the subject GBGVs (height measured at the withers) was provided by the owners. Dogs with heights of 38 to 46 cm were classified as short-legged (the breed standard) and dogs taller than 58 cm were classified as long-legged. All dogs fell into one of these two size classes. All samples and phenotype information were provided by the owners with informed consent. The study protocols were approved by the University of Missouri Animal Care and Use Committee.

### 2.2. Molecular Genetics

DNA from each dog was isolated from EDTA anticoagulated blood or FTA Elute cards as previously described [8,9]. Litters born to the short-legged parents of each tall dog included both tall and short-legged dogs (Figure 1 and Figure 2). DNA samples were genotyped for the *FGF4RG* chromosome 12 and 18 insertions using 3-Primer PCR amplification. Flanking primers were used in separate reactions for each insertion site as previously described [7]. In each assay, one pair of the primers flanking the insertion site amplified the non-insertion allele. A third *FGF4RG* internal primer was paired with one of the flanking primers to amplify a segment of the *FGF4RG* insertion allele. This 3-primer assay generated different product sizes depending on whether the retrogene insertion was absent or present. The PCR products were visualized using a microcapillary system (QIAxcel, QIAGEN N.V., Venlo, The Netherlands). Sanger sequencing was used to confirm the identities of the amplicons. Most Dachshunds and Basset Hounds have both the chromosome 12 and 18 insertions, whereas neither insertion has been reported in Great Danes. Thus, DNA from Dachshunds and a Basset Hound were used as positive controls for the genotyping assays, and DNA from a Great Dane was used as a negative control.

### 2.3. Statistical Analysis

Dog heights measured at the withers were compared between the long-legged and short-legged groups with a *t*-test. Pearson chi square analysis was performed to assess the association between *FGF4RG* genotypes and leg length phenotype. For these analyses, dogs of both sexes were pooled. Dogs were each assigned to one of six groups for the statistical analyses: short-legged/homozygous for the insertion allele; short-legged/heterozygous the insertion allele; short-legged/homozygous for the non-insertion allele; long-legged/homozygous for the insertion allele; long-legged/heterozygous the insertion allele; long-legged/homozygous for the non-insertion allele. SigmaPlot v16.0 (Grafiti Software, Palo Alto, CA, USA) was utilized for statistical analyses.

## 3. Results

The dogs evaluated in the study fell into two height classes; short-legged dogs were an average of 41 ± 2 cm tall and long-legged dogs were an average of 59 ± 1 cm tall (difference *p* < 0.0001). Of the 41 GBGVs that had short legs, 14 were homozygous for the chromosome 18 insertion allele and 27 were heterozygous for the *18-FGF4RG* insertion. There was no difference in the average height between the heterozygous and homozygous dogs. All 6 of the tall dogs were homozygous for the non-insertion allele (Table 1). The association between *18-FGF4RG* genotype and leg length phenotype was statistically significant (*p* < 0.01). None of the GBGVs that were evaluated had the *12-FGF4RG* insertion that is common in other breeds with short legs. The short-legged Dachshunds were homozygous for the *12-FGF4RG* and *18-FGF4RG* insertions, a Basset Hound was heterozygous for the *12-FGF4RG* insertion and homozygous for the *18-FGF4RG* insertion, and a Great Dane of normal breed stature was homozygous for the chromosomes 12 and 18 non-insertion alleles.

## 4. Discussion

The domestication of dogs began over 30,000 years ago with the association between wolves and human hunter-gatherers [10,11]. Early breeding selection was based primarily on behavioral traits that fostered compatibility between ancient dogs and their human companions. Natural variation and selective breeding led to the development of distinct breeds at least as long as 9500 years ago [11,12]. Over the millennia since their first domestication, numerous other dog breeds were established by selectively breeding dogs with distinct phenotypic features that appeared as a result of natural variation. In the modern era breed standards based primarily on physical features and behavioral traits have become formalized by dog breed organizations. Although variation between breeds is considered desirable, uniformity within a breed is considered the ideal. Among dog fanciers, dogs that most closely meet the breed physical standards are used most widely as breeders. Modern breeding practices still rely primarily on phenotypic selection. However, with advances in molecular genetic analyses, DNA sequence variants associated with many specific phenotypic traits have been identified, enabling the use of DNA sequence analysis to guide breeding selection.

In an individual dog with generally desirable phenotypic features, an undesired natural phenotypic variant of a particular trait may occur. In such instances, a breeder may wish to preserve the desirable phenotypic features but select against the single undesired variant feature. For GBGVs, breeding selection is performed to maintain the short-legged phenotype. If selective breeding is based on phenotype alone, breeders may elect to discontinue breeding dogs that have produced long-legged offspring. This would constrict the breeding population resulting in increased risk of homozygosity for deleterious genetic variants. Now, with the ability to screen for the *18-FGF4RG* insertion, breeders can avoid producing long-legged puppies by breeding dogs that are heterozygous to dogs that are homozygous for the insertion or by breeding long-legged dogs to dogs that are homozygous for the insertion. By increasing the options for selecting breeding pairs, this will enable maintaining the short-legged phenotype while reducing the risk of producing puppies with recessive genetic disorders.

*18-FGF4RG* retrogene insertion is associated with short stature (short-leggedness) in multiple other dog breeds [1]. In most of these breeds, all of the dogs evaluated were homozygous for the *18-FGF4RG* insertion. On the other hand, the insertion allele was not present in tall (long-legged) breeds. Among the short-legged breeds in which all dogs evaluated were homozygous for the insertion allele were Basset Hound, Cardigan and Pembroke Welsh Corgi, Dachshund, Pekingese, Petit Basset Griffon Vendéen (PBGV), Shi Tzu, Skye Terrier, Swedish Valhund, Tibetan Spaniel, and West Highland Terrier [1]. Homozygosity for the *18-FGF4RG* insertion has also been reported in other short-legged breeds including Cairn Terrier and Coton de Tulear, as well as in short-legged mixed breed dogs [7]. Although there is an association of *18-FGF4RG* insertion genotype and leg length among many breeds, this alone does not establish a cause-and-effect relationship. Of four Grande Basset Griffon Vendéen (GBGV) that were evaluated previously, three were heterozygous for the *18-FGF4RG* insertion, and one was homozygous for the insertion [1]. This suggested that if the *18-FGF4RG* insertion was responsible for the short-legged phenotype, one copy of the retrogene insertion was sufficient to confer short-leggedness. However, because no dogs that were either long-legged or that lacked the insertion were identified, the data did not establish that the presence of the insertion was responsible for short-leggedness in this breed. We found that among 22 GBGVs born between 2010 and 2023, all 6 that were long-legged lacked the *18-FGF4RG* insertion, whereas all of the dogs with short legs had either one or two copies of the insertion. This makes it highly probable that the presence of either one or two copies of the insertion conferred the short-legged phenotype.

The finding that 6 of the 47 GBGVs that were evaluated were homozygous for the *18-FGF4RG* insertion and 27 were heterozygous indicates that despite many generations of breeding in which the short-legged phenotype was selected for, the genetic variant responsible for long-leggedness is still common in the breed. This is due in large part to the fact that both dogs homozygous and heterozygous for the insertion exhibit the short-legged phenotype. The evidence that the long-legged phenotype in GBGVs is due to lack of the *18-FGF4RG* insertion allows breeders to utilize genotyping for the insertion allele to distinguish between dogs heterozygous and homozygous for the insertion and thus minimize the frequency with which long-legged puppies are produced. The high frequency of the non-insertion allele in the cohort evaluated in this study may not be representative of the entire breed. Participation in the study was voluntary and breeders whose dogs had produced long-legged offspring may have been more motivated to submit samples than others.

Long-legged variants associated with the absence of the *18-FGF4RG* insertion may also appear in other breeds that normally have short legs. For example, it was recently reported that two Wirehaired Dachshund littermates with abnormally long legs were born to parents with the short-legged phenotype typical of the breed [6]. Both long-legged dogs lacked the *18-FGF4RG* insertion, whereas both parents and a short-legged littermate were heterozygous for the insertion. The Dachshund group of breeds have been established for centuries, and the short-legged phenotype associated with the *18-FGF4RG* insertion appears to be fixed in the breed. Thus, the appearance of Dachshunds lacking the insertion suggests that retrogene can be lost even in breeds where it has been maintained for many generations. Therefore, in many breeds characterized by short-leggedness, the rare appearance of long-legged puppies is likely to be due to loss of the *18-FGF4RG* insertion in prior generations.

In addition to insertion of the *FGF4RG* retrogene on chromosome 18, many dog breeds have an *FGF4RG* retrogene insertion on chromosome 12 [7,13,14]. The *12-FGF4RG* insertion is associated with intervertebral disc disease [13] and in at least some of breeds, it has also been found to be associated with height (leg length). For example, among Nova Scotia Duck Tolling Retrievers dogs homozygous for the *12-FGF4RG* insertion were significantly shorter than dogs lacking the insertion, whereas heterozygous dogs were of intermediate height [7]. None of the GBGVs evaluated for the current study harbored the *12-FGF4RG* insertion, so it appears that the height differences were due exclusively to differences in *18-FGF4RG* genotype.

In addition to *FGF4RG*, numerous other retrogene insertions have been identified in the canine genome. For example, 3892 candidate retrocopies from 1316 parental genes were identified in the CanFam4 genome assembly [15], and 3025 retrocopies including 476 intact retrogenes, 2518 retropseudogenes, and 31 chimerical retrogenes were identified in the CanFam3.1 genome assembly [16]. Unlike the *FGF4* retrogenes, the potential phenotypic effects of most of these retrogenes, if any, are unknown. However, in human subjects retrogene expression has been associated with a number of hereditary disorders [17,18]. Thus, it seems likely that at least some of the other retrogenes in the canine genome are expressed and have phenotypic effects. Over 90% of the retrocopy insertions in the CanFam4 were also present in the wolf genome assembly, suggesting that they arose long ago. Based on the number of generations since genome divergence, it was estimated that new retrocopy insertions appear, on average, in 1 out of 3514 births. Like the *FGF4* retrogene, formation of these retrogenes can lead to new phenotypic features that can be benign, beneficial, or pathogenic [16].

The mechanism by which the presence of the *18-FGF4RG* insertion confers short-leggedness has not been elucidated, but it has been established in other dog breeds that this retrogene is expressed, and it has been hypothesized that abnormal excess production of fibroblast growth factor 4 (FGF4) in chondrocytes during development impairs limb elongation [1]. Consistent with this hypothesis, it has been demonstrated that FGF4 plays a role in axial elongation during development in mice [19]. Because the FGF4 retrogene does not contain the introns of the parent gene and because its location in the genome is different from that of the parent gene, it is unlikely that expression of the retrogene is under the same regulatory control as the parent gene. Aberrantly regulated expression of FGF4 from the *18-FGF4RG* retrogene during development is therefore likely to account for the short-leggedness in dogs. It is interesting that no other phenotypic effects of the retrogene expression have been recognized. In contrast to the *18-FGF4RG* insertion, an *FGF4RG* retrogene insertion on canine chromosome 12 is associated not only with leg length but also with intervertebral disc disease [7,13,14]. This suggests that regulation of expression of the retrogene varies depending on its location within the genome. It should be possible to investigate this possibility in a cell culture model.

Retrogene insertions can have phenotypic effects even if the retrogene is not expressed. For example, it was found that insertion of a *SNNL1* retrocopy on canine chromosome 18 likely disrupted regulation of a nearby gene involved in coat color determination in Poodles [20]. Although the roles of most retrogenes, if any, remain to be elucidated, analysis of retrogenes across multiple species indicates that they are subject to natural selection, and those that survive over the course of evolution are likely to be involved in adaptation [21]. The situation with *18-FGF4RG* is a case where retrogene survival is determined primarily by human-directed selection rather than natural selection. In the future, identification of the effects of other retrogene insertions on canine phenotypes can be employed to select for desired traits or eliminate undesired traits, including some hereditary disorders. 

## 5. Conclusions

In summary, *18-FGF4RG* is the main determinant of increased height (leg length) by an average of more than 35% above the breed standard in GBGVs. The expression of the retrogene during development is likely primarily responsible for the standard short-legged phenotype in dogs with one or two copies of the insertion. There was moderate variability in leg length among both the short- and long-legged dogs, which suggests that there are other determinants that have lesser effects on height in this breed. To avoid generating dogs substantially taller than the breed standard, dogs can be genotyped for the insertion allele before breeding, rather than relying solely on phenotypic selection, which does not distinguish between dogs heterozygous or homozygous for the insertion allele.

## Figures and Tables

**Table 1:** Association between *18-FGF4RG* insertion genotype and height phenotype in GBGVs.

18-FGF4RG Insertion	Short	Tall
Insertion/Insertion	14	0
Insertion/No Insertion	27	0
No Insertion/No Insertion	0	6

## Data Availability

The raw data supporting the conclusions of this article will be made available by the authors on request.

## Abbreviations

The following abbreviations are used in this manuscript:

GBGV	Grand Basset Griffon Vendéen
PBGV	Petit Basset Griffon Vendéen
EDTA	Ethylene diamine tetra acetic acid
PCR	Polymerase chain reaction
I	Insertion
N	Non-insertion
FGF4	fibroblast growth factor 4
